# A Cohort Study of the Impact of Tooth Loss and Periodontal Disease on Respiratory Events among COPD Subjects: Modulatory Role of Systemic Biomarkers of Inflammation

**DOI:** 10.1371/journal.pone.0068592

**Published:** 2013-08-08

**Authors:** Silvana P. Barros, Robert Suruki, Zvi G. Loewy, James D. Beck, Steven Offenbacher

**Affiliations:** 1 Department of Periodontology, School of Dentistry, University of North Carolina, Chapel Hill, North Carolina, United States of America; 2 Worldwide Epidemiology, GlaxoSmithKline R&D, Research Triangle Park, North Carolina, United States of America; 3 GlaxoSmithKline Consumer Healthcare, Parsippany, New Jersey, United States of America; 4 Department of Dental Ecology, School of Dentistry, University of North Carolina, Chapel Hill, North Carolina, United States of America; Clinica Universidad de Navarra, Spain

## Abstract

**Background:**

In COPD patients, fatal and non-fatal respiratory-related events are influenced by age, severity of respiratory disease, and comorbidities.

**Objectives:**

Analyze the effects of edentulism, periodontal disease and systemic biomarkers of inflammation on the occurrence of serious fatal and non-fatal respiratory-related events among subjects with COPD.

**Methods:**

Cases were identified from Dental Atherosclerosis Risk in Communities study. Edentulism was defined as study participants without any natural teeth or implants. Participants with one or more natural teeth (comprising 11,378 subjects) were studied as dentate subjects. Periodontal disease status among dentate individuals was determined using the consensus definitions published by the joint Center for Disease Control/American Association of Periodontology working group). Adjusted Hazard Models are developed to evaluate the relationship between edentulism/periodontal disease and COPD Related Events. Models were then stratified by GOLD Stage I, II and III/IV. Serum biomarkers were also evaluated to explore the effect of systemic inflammation.

**Results:**

A statistically significant association was found between oral health status and COPD-related events, even adjusting for conditions such as hypertension, smoking and diabetes. Edentulous individuals who had been diagnosed with COPD had a higher incidence and were at greater risk of having a COPD related event (hospitalization and death) than individuals who had teeth and whose mouths had healthy periodontal status. However, being edentulous did not convey excess risk for COPD-related events for those study participants who were classified as GOLD III/IV at baseline. Finally, we showed that individuals who had levels of serum IL-6 in the highest two quartiles were at even higher risk for COPD-related events.

**Conclusions:**

These findings suggest that the risk for COPD-related events after adjusting for potential confounders may be attributable to both edentulism and elevated serum IL-6 levels.

## Introduction

Non-Communicable Diseases (NCDs) including chronic respiratory diseases, together with diabetes, cancer and cardiovascular diseases, are the leading cause of disease burden and mortality in the world. Chronic obstructive pulmonary disease (COPD) is the fifth leading cause of death world-wide and the third leading cause of death in the United States, affecting as many as 24 million Americans and resulting in 700,000 hospital admissions, and 124,000 deaths annually [1]. COPD, which is considered the major respiratory NCD with a 10.1% prevalence of stage II or higher COPD has been associated with low-grade systemic inflammation [2], [3]. Thus, although COPD is primarily characterized by airflow limitation that is generally associated with an abnormal inflammatory response in the lungs, it is also often associated with a significant systemic inflammatory response which has been correlated with adverse clinical effects [4].

The link between oral infection and various respiratory diseases in the dentate population has been the focus of several studies [5], [6], more recently, Preshaw et al [7] presented a review focused on the association of oral and systemic health as it relates to health-related quality of life .

Data from our research group demonstrated a significant association between prior COPD and edentulism [8] . This observation correlates well with a study that resulted in the identification of diverse microbes, including respiratory pathogens on the surfaces of dentures [9].

In addition to its association with an inflammatory response of the lung, COPD is also associated with systemic inflammation, including systemic oxidative stress, activation of circulating inflammatory cells and increased levels of inflammatory cytokines [9]. Levels of inflammatory biomarkers such as C-reactive protein (CRP), Interleukin-6 (IL-6) and sICAM-1 have been reported to be elevated in COPD [10], [11], [12]. By improving our understanding of this association with the potential for reducing the incidence of COPD through treatment of periodontal diseases, which would have important public health and clinical implications, in the present study we focused on analyzing the effects of edentulism, periodontal disease and systemic biomarkers of inflammation on the occurrence of serious fatal and non-fatal respiratory-related events among subjects with COPD. The study population was that of the Atherosclerosis Risk in Communities (ARIC) Study [13].

## Methods

### Study Population

The ARIC study included 15,792 individuals aged 45 to 64 years selected from four U.S.A. communities. This prospective cohort study has been previously described in greater detail. (The ARIC Investigators, 1989). For the current study, data from individuals participating in the Dental Atherosclerosis Risk in Communities (D-ARIC) study were also used to examine the associations between edentulism, periodontal disease, and systemic biomarkers of inflammation, and the risk of serious fatal and nonfatal COPD-related events. The D-ARIC was a cross-sectional investigation conducted in a subset of dentate participants at Visit 4 (1996–1998). Eligible participants were all aged 45–64 at study entry (i.e., Visit 1) and had spirometry measurements at Visit 2 (3 years later) and periodontal examinations at D-ARIC Visit 4 (6 years from visit 2). Edentulous individuals at Visit 4 were also eligible for inclusion. Lastly, all individuals were required to have at least 1 year of follow-up subsequent to Visit 4.

### COPD Definition

Based on spirometry measurements obtained at Visit 2, individuals were considered to have COPD if they had a ratio of forced expiratory volume in 1 second (FEV1) to forced vital capacity (FVC) less than 0.7. Subsequently, individuals with COPD were further categorized by disease severity, using the spirometry-based criteria for severity outlined in the Global Initiative for Chronic Obstructive Lung Disease (GOLD) guidelines (Table 1). (Global Initiative for Chronic Obstructive Lung Disease (GOLD), 2007).

**Table 1:** Spirometric Classification of COPD Severity Based on Post-Bronchodilator FEV1.

GOLD Stage	Spirometry Criteria
Stage I	FEV1/FVC <0.7 and FEV1 ≥80% predicted
Stage II	FEV1/FVC <0.7 and 50% ≤ FEV1 <80%
Stage III	FEV1/FVC <0.7 and 30% ≤ FEV1 <50%
Stage IV	FEV1/FVC <0.7 and FEV1 <30%

### COPD-Related Event

The primary endpoint for this study was the occurrence of COPD-related events (e.g., hospitalization for COPD exacerbation) or COPD-related death during the 5-year period following Visit 4. In order to be considered related to COPD, a hospitalization was required to have an ICD9 code for COPD (491.xx, 492.x, or 496.x) entered in the discharge notes. Similarly, a death was considered COPD-related if any of the above listed ICD9 codes were included as a primary cause, secondary cause or underlying cause.

### Edentulism and Periodontal Disease

Edentulism cases were defined as study participants without any natural teeth or implants. Participants with one or more natural teeth (comprising 11,378 subjects) were selected as dentate subjects. Periodontal disease status among dentate individuals was determined using the consensus definitions published by the joint Center for Disease Control/American Association of Periodontology/(CDC/AAP) working group. Periodontal disease severity was determined based on clinical attachment level (CAL) and probing depth (PD) according to the criteria described in Table 2.

**Table 2:** Periodontal Disease Severity defined by clinical parameters.

	Clinical Definition		
Disease Category	CAL		PD
Severe periodontitis	≥2 interproximal sites with CAL ≥6 MM (not on same tooth)	And	≥1 interproximal site with PD ≥5 mm
Moderate periodontitis (i.e., Entry)	≥2 interproximal sites with CAL ≥4 mm (not on same tooth)	Or	≥2 interproximal sites with PD ≥5 mm (not on same tooth)
No or mild periodontitis (i.e., including healthy and gingivitis)	Neither “moderate” nor “severe” periodontitis		

### Systemic Immune Mediators

Interleukin-6 (IL-6), C-reactive protein (CRP), and soluble intercellular adhesion molecule 1 (sICAM-1) concentrations from once thawed serum aliquots (frozen at −80C from collection until December, 2009) were all measured by ELISA techniques. Spectrophotometric endpoints were determined on a SpectraMax M2 plate reader (Molecular Probes, Sunnyvale, CA) using reagent assay kits from R&D Systems (Minneapolis, MN) according to manufacturer's instructions. The Softmax® (v.5.0.1) control software package was used to fit the standard curve data using either 4-PL or 5-PL fitting algorithms to provide a best fit of the seven-point (duplicate) standard curve after subtraction of the mean reagent blank values from all measured optical densities. Standard curve concentrations ranged from 0.156–10 pg/ml for serum IL-6, 780–50,000 pg/ml for CRP, and 1,560–50,000 pg/ml for sICAM-1. Subsequently, the immune mediator concentrations were computed by application of the standard curve fitting equation.

### Other Covariates

Participants of this study were additionally characterized with respect to research center (Jackson, Mississippi; North Carolina; Washington; Minnesota), race (African American, Caucasian), age, sex, and body mass index. History of diabetes and hypertension were also included in the analysis, as well as income and education as measures of socioeconomic status. Self-reported smoking data (both cigarettes and cigars) collected at Visit 4 was examined as a categorical variable (e.g., current, former, and never). Additionally, pack-years of smoking were calculated for current and former smokers.

### Statistical Analysis

Patient characteristics were compared between those with and without a COPD related event using chi-squared tests and t-tests for categorical variables and continuous variables, respectively. Kaplan-Meier curves were constructed for each periodontal disease and edentulous status patient group to examine the time to COPD related event; differences between Kaplan-Meier curves were compared using the log-rank test. Cox proportional hazards regression was used to compute hazards ratios (HR) and 95% confidence intervals for COPD-related events. In addition to the crude model, a minimally adjusted and a fully adjusted model were also specified; covariates were selected for inclusion into the model a priori or based on bivariate analyses. To measure modification due to COPD severity, the above models were also stratified by COPD GOLD Stage (I, II, III/IV). All analyses were performed using the SAS software, version 9.1 (SAS Institute Inc, Cary, NC). A two-sided *P* value of <0.05 was considered statistically significant.

## Results

**Figure 1:**
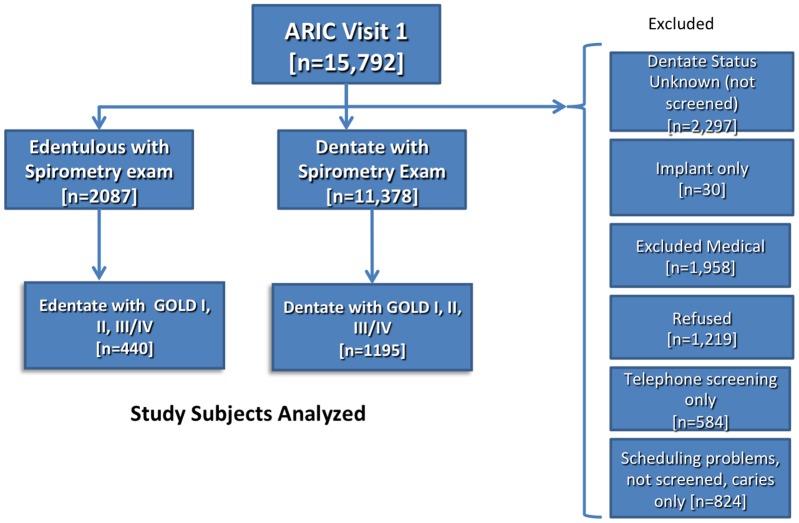
Study Subjects Analyzed.

The purpose of this investigation was to determine the effects of edentulism (total loss of teeth), periodontal disease and systemic biomarkers of inflammation on COPD events in a cohort population with spirometry-identified COPD. The participant flow diagram with explanations for inclusion and exclusion appears in Figure 1. Among the 15,792 ARIC participants there were a total of 1635 subjects with GOLD stage I,II or III/IV COPD with known dentate status (with or without teeth) and full-mouth periodontal examination data for those with teeth. There were a total of 440 edentulous subjects and 1195 dentate subjects who were followed for events over a 5 year period with an overall event rate of 24.4% as shown in Table 3. Oral health status was significantly related to events (p<0.0001) with event rates demonstrating a gradient as related to worsening of oral health from a rate of 10.5% among dentate, periodontally healthy individuals increasing to 23.8% among those with severe periodontal disease with the highest rate of COPD events among those who have lost all teeth due to oral diseases (43.9%). As expected, there was an increased event rate with increased GOLD stage, p<0.0001 with those having GOLD Stage III/IV disease experiencing approximately 3.4 times the event rate as those with GOLD stages I–II. COPD subjects who experienced events were more likely to be Caucasian, younger, male, diabetic, hypertensive, tobacco user (current or former), low income and less educated.

**Table 3:** Potential Explanatory Variables by COPD Related Events.

	Events (No)	Events (Yes)	p-value
	1236 (75.6%)	399 (24.4%)	
Perio/Edent			
Health	365 (89.5%)	43 (10.5%)	
Entry	403 (81.1%)	94 (18.9%)	
Severe	221 (76.2%)	69 (23.8%)	
Edentulous	247 (56.1%)	193 (43.9%)	<0.0001
Gold			
I	763 (87.6%)	108 (12.4%)	
II	413 (64.0%)	232 (36.0%)	
III–IV	60 (50.4%)	59 (49.6%)	<0.0001
Center			
Jackson	166 (83.4%)	33 (16.6%)	
NC	365 (70.7%)	151 (29.3%)	
Wash	350 (72.8%)	131 (27.2%)	
Minn	355 (80.9%)	84 (19.1%)	<0.0001
African-American	197 (82.4%)	42 (17.6%)	
Caucasian	1035 (74.4%)	356 (25.6%)	0.008
Age Mean (StdDev)	66.0 (5.13)	63.9 (5.69)	<0.0001
BMI Mean (StdDev)	27.1 (5.43)	27.3 (4.83)	0.36
Male	669 (71.9%)	261 (28.1%)	
Female	567 (80.4%)	138 (19.6%)	<0.0001
Diabetic (Yes)	159 (65.7%)	83 (34.3%)	
(No)	1072 (77.3%)	214 (22.7%)	<0.0001
Hypertension (Yes)	393 (69.6%)	172 (30.4%)	
(No)	833 (78.7%)	225 (21.3%)	<0.0001
Smoking			
Never	403 (94.6%)	23 (5.4%)	
Former	587 (74.3%)	203 (25.7%)	
Current	239 (58.6%)	169 (41.4%)	<0.0001
Pack Years Mean (StdDev)	22.4 (24.8)	47.0 (27.2)	<0.0001
Income			
Low	368 (69.3%)	163 (30.7%)	
Medium	444 (75.3%)	146 (24.8%)	
High	373 (83.6%)	73 (16.4%)	<0.0001
Education			
Basic	224 (61.9%)	138 (38.1%)	
Intermediate	536 (77.0%)	160 (23.0%)	
Advanced	475 (82.6%)	100 (17.4%)	<0.0001

**Figure 2:**
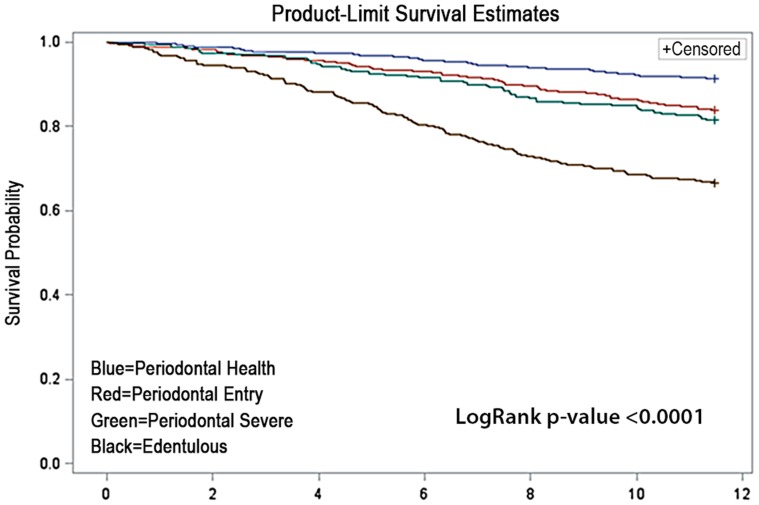
Product-Limit Survival Estimates.

The unadjusted Kaplan Meier Curve showing the event rates for COPD hospitalization/death for all subjects with GOLD stages of I, II and III/IV appear in Figure 2. As suggested by the dentate status and periodontal disease data in Table 3, the survival function clearly separated periodontal health from edentulous individuals, while those with Entry and Severe forms of periodontal disease exhibited intermediate survival functions. Overall, the oral health status has a dramatic effect on unadjusted event-free survival rates among COPD subjects with a log rank p-value of <0.0001. However, the effects of adjustment for relevant covariates and confounders on these trends are shown in Table 4. Cox proportional hazards models using the periodontally healthy dentate group as the referent population in unadjusted models demonstrate a clear trend and a gradient with increasing periodontal disease severity increasing the hazards ratios (HR) from 1.90 (95% confidence interval: 1.28–2.83) to 2.22 (1.44–3.41) and 4.66 (3.21–6.78) for edentulism. After adjustment for relevant confounders from Table 1 including age, race, center, gender and smoking, only edentulism remains a significant risk factor for events with a hazard ratio of 2.45 (1.61–3.74). Inclusion of additional variables in the final model did not contribute to the overall fit of the model.

**Table 4:** Hazard Ratios for COPD Related Events.

	Hazard Ratio (95% Conf. Limit)	
	Unadjusted	Adjusted*
Periodontal Health	Ref	Ref
Periodontal Entry	1.90 (1.28–2.83)	1.37 (0.89–2.12)
Periodontal Severe	2.22 (1.44–3.41)	1.34 (0.83–2.18)
Edentulous	4.66 (3.21–6.78)	2.28 (1.46–3.56)

* Adjusted for Race/Center (5-levels), Gender and Age, Smoking (3-levels), Pack Years, Hypertension, Education (3-levels) and BMI.

The effects of oral health status on COPD-related events are stratified by GOLD stage in Table 5. In unadjusted models for GOLD stage I subjects there is an apparent trend of increasing hazard ratio associated with periodontal disease severity with the highest risk observed in the edentulous individuals [HR = 5.06 (2.60–9.85)]. However, after adjustment for center, race, gender, age, smoking, education and BMI, only edentulism remained statistically significant albeit with attenuated risk [HR = 2.37 (1.03–5.45)]. The effect of oral disease on events for subjects with GOLD stage II demonstrated a similar pattern as GOLD I subjects, with a comparable magnitude of risk [HR = 2.12 (1.17–3.83)] for edentulous subjects. There was no association between oral status and events among GOLD stage III/IV subjects (Table 5).

**Table 5:** Hazard Ratios for COPD Related Events Stratified by GOLD Stage.

	Hazard Ratio (95% Conf. Limit)	
	Unadjusted	Adjusted*
GOLD I		
Periodontal Health	Ref	Ref
Periodontal Entry	2.38 (1.21–4.68)	1.85 (0.86–4.01)
Periodontal Severe	2.49 (1.18–5.26)	1.53 (0.64–3.63)
Edentulous	5.06 (2.60–9.85)	2.37 (1.03–5.45)
GOLD II		
Periodontal Health	Ref	Ref
Periodontal Entry	1.60 (0.91–2.81)	1.17 (0.64–2.13)
Periodontal Severe	2.02 (1.12–3.64)	1.34 (0.70–2.58)
Edentulous	3.51 (2.08–5.94)	2.12 (1.17–3.83)
GOLD III/IV		
Periodontal Health	Ref	Ref
Periodontal Entry	0.69 (0.24–1.98)	0.69 (0.19–2.54)
Periodontal Severe	0.65 (0.13–3.22)	0.15 (0.02–1.35)
Edentulous	1.39 (0.56–3.44)	0.62 (0.16–2.39)

* Adjusted for Race/Center (5-levels), Gender and Age, Smoking (3-levels), Pack Years, Hypertension, Education (3-levels) and BMI.

Serum biomarkers of inflammation including IL-6, CRP and sICAM-1 have been associated with periodontal disease and COPD morbidity. Table 6 and 7 explore the potential role of these systemic mediators as intermediary explanatory variables that link the observed association between oral disease and COPD morbidity. Subjects with higher levels of serum IL-6, CRP or sICAM had higher event rates (Table 6). For example, subjects with the lowest levels of IL-6 (lowest quartile) experienced lower event rates (7.5%) as compared to subjects in the highest quartile of serum IL-6 (41.8%). All three biomarkers were associated with statistically significantly higher event rates when elevated in the highest quartile; however, these significant trends are unadjusted. The models that include oral health status and serum IL-6 levels by quartiles, and another that includes oral health status and quartile of serum CRP are shown in Table 7. Models that include oral health status and sICAM are not shown because no significant associations with events were observed. Table 7 displays unadjusted and fully adjusted Models. These Cox Proportional Hazards models include both oral disease status using Periodontal Health as the referent group and the lowest quartile of IL-6 or CRP as the referent group. In the unadjusted IL-6 model severe periodontal disease and edentulism as well as all three of the upper quartiles of IL-6 remain significantly associated with increased HR. There is a gradient response noted with increasing IL-6 levels. In the fully adjusted models severe periodontal disease is no longer significantly associated with increased risk, but edentulism remains significant [HR = 2.66 (1.34–5.29)] as well as serum IL-6 levels [upper quartile [(HR = 3.37 (1.49–7.63)]) within the same models, suggesting independent but approximately comparable effect size on the hazard rate. A similar pattern is present for the effects of oral health status on events when including serum levels of CRP, but CRP level was not a significant effect modifier in adjusted models. Thus, among the three inflammatory biomarkers serum IL-6 has a significant interaction with oral health status to increase the hazard rate for events when serum IL-6 levels are elevated. For example, among edentate subjects with highest quartile of serum IL-6, the Hazard ratio for COPD related events was 8.9. However, based upon the data shown in Table 5, the effects of oral disease and serum IL-6 on morbidity would not be expected to be evident for all COPD GOLD Stages.

**Table 6:** Quartiles of Serum Biomarkers by COPD Related Events.

	Event (No)	Event (Yes)	p-value
Serum IL-6			
Quartile 1	135 (92.5%)	11 (7.5%)	
Quartile 2	113 (77.4%)	33 (22.6%)	
Quartile 3	102 (69.9%)	44 (30.1%)	
Quartile 4	85 (58.2%)	61 (41.8%)	<0.0001
Serum CRP			
Quartile 1	103 (83.7%)	20 (15.5%)	
Quartile 2	96 (79.3%)	25 (20.7%)	
Quartile 3	84 (68.9%)	38 (31.2%)	
Quartile 4	77 (62.6%)	46 (37.4%)	0.0005
Serum sICAM-1			
Quartile 1	115 (78.8%)	31 (21.2%)	
Quartile 2	121 (82.9%)	25 (17.1%)	
Quartile 3	101 (69.2%)	45 (30.8%)	
Quartile 4	98 (67.1%)	48 (32.9%)	0.004

**Table 7:** Hazard Ratios for COPD Related Events adding Serum Biomarkers.

	Hazard Ratio (95% Conf. Limit)	
	Unadjusted	Adjusted*
Oral Condition + Serum IL-6		
Periodontal Health	Ref	Ref
Periodontal Entry	..	..
Periodontal Severe	1.92 (1.03–3.55)	1.69 (0.85–3.36)
Edentulous	3.91 (2.20–6.95)	2.66 (1.34–5.29)
Serum IL-6 Q1	Ref	Ref
Serum IL-6 Q2	2.69 (1.20–6.03)	2.12 (0.92–4.90)
Serum IL-6 Q3	3.85 (1.77–8.39)	3.14 (1.40–7.02)
Serum IL-6 Q4	5.17 (2.41–11.1)	3.37 (1.49–7.63)
Oral Condition + Serum CRP		
Periodontal Health	Ref	Ref
Periodontal Entry	..	..
Periodontal Severe	2.31 (1.18–4.54)	1.65 (0.78–3.52)
Edentulous	5.04 (2.67–9.50)	3.17 (1.51–6.66)
Serum CRP Q1	Ref	Ref
Serum CRP Q2	0.91 (0.47–1.79)	0.80 (0.40–1.60)
Serum CRP Q3	1.50 (0.82–2.75)	1.40 (0.73–2.66)
Serum CRP Q4	1.75 (0.97–3.14)	1.14 (0.61–2.15)

**Figure 3:**
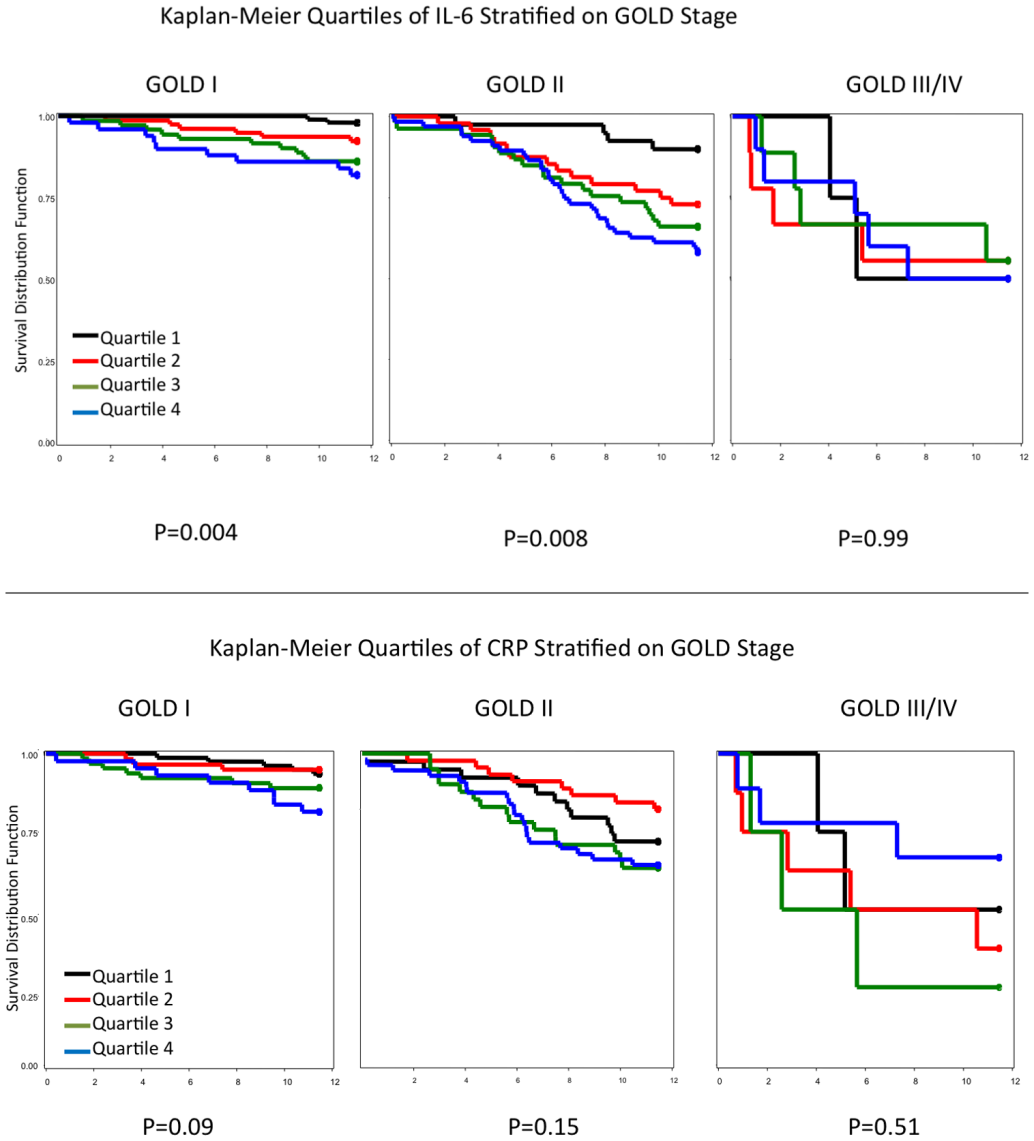
Kaplan-Meier Quartiles of IL-6 Stratified on GOLD Stage.

Figure 3a illustrates the Kaplan-Meier survival function for quartiles of serum IL-6 stratified by GOLD Stage. The effects of elevated IL-6 on COPD events is evident among subjects with GOLD stage I and GOLD stage II, but not for GOLD stage III/IV. A significant gradient for increased risk for events with increasing IL-6 levels (by quartile) was observed in subjects with GOLD stage I and II disease (p = 0.004 and p = 0.008, respectively). No effects are significant for CRP levels for any of the GOLD stages in Figure 3B. These data suggest that serum levels of IL-6 independently contribute to COPD-related events among those with GOLD stage I and GOLD stage II disease.

## Discussion

In this population-based longitudinal study, we observed a statistically significant association between oral health status and COPD-related events, even adjusting for conditions such as hypertension and diabetes. Spirometry values were used to determine GOLD stage as described in the Methods section. As shown in Table 1, incidence of COPD-related events had a monotonic positive association with GOLD stage ranging from 12.4% for individuals with a GOLD stage of I to 49.6% for those with a GOLD stage of III/IV.

### Edentulism and COPD-Related Events

We found that edentulous (total loss of teeth) individuals who had been diagnosed with COPD had a higher incidence and were at greater risk (HR = 2.28 and 95% CI = 1.46–3.56) of having a COPD related event (hospitalization and death) than individuals who had teeth and whose mouths had healthy periodontal status (tables 1 and 2). This excess risk was independent of the effects of age, race, sex, field center, smoking, pack years, hypertension, education, and BMI. In additional, fully adjusted models, we found that being edentulous conveyed excess risk for COPD-related events even when stratified by GOLD score for individuals who were classified as GOLD stage I or GOLD stage II at baseline (Table 3). However, being edentulous did not convey excess risk for COPD-related events for those study participants who were classified as GOLD III/IV at baseline. Finally, we showed that edentulous individuals who had levels of serum IL-6 in the highest two quartiles were at even higher risk for COPD-related events. These findings suggest that the risk for COPD-related events after adjusting for potential confounders may be attributable to both edentulism and elevated serum IL-6 levels.

There are a number of potential mechanisms that may underlie this association and additional factors that may account for the findings. Because COPD is associated with an abnormal inflammatory response of the lung parenchyma to inhaled pollutants and gases, an apparent explanation for the detection of systemic inflammation on these patients was a hypothesis that systemic inflammation in COPD was originating in a form of “spillover” of the pulmonary compartment, which has not been proven [14].

However one reason for our finding that edentulism is predictive of COPD-related events may be that more than 97% of the study participants who had lost all of their teeth have dentures and it is known that biofilm that forms on dentures can house bacteria, yeasts and fungus that result in inflammatory response from the oral tissues. Similar to the biofilm on natural teeth, denture biofilm is complex due to the types and numbers of organisms it contains, as well as its organized structure. Recently Glass et al. reported on the complex nature of the microbial flora contained within the denture biofilm, identifying over 900 individual species of aerobic and anaerobic bacteria, yeasts and amoebae [9]. Others have reported similar complexities for the microbial content of denture plaque [15]. It has also been long recognized that unlike the dental biofilm, the biofilm that forms on denture materials harbors a much larger population of yeasts, and *Candidal* yeasts, and specifically *Candida albicans*, have been shown to have strong pathogenic associations with the presence of denture stomatitis [15], [16], [17], [18], [19], [20] .

Since periodontal disease does lead to major tooth loss in a subset of the population that have a strong inflammatory response to infection, it is likely that infections on dentures will lead to a similar inflammatory response. Thus, those study participants who lost their teeth due to periodontal disease and have an inflammatory response to the pathogens/fungus may be hyper-inflammatory responders who are more susceptible to having incident COPD-related events that lead to hospitalization and death. Since reduction of most bacteria related to periodontal disease is expected after full mouth tooth extraction, these individuals may have reduced their systemic levels of inflammation due to periodontal infections [21]. However, they may have increased their overall risk for COPD related events via denture biofilm accumulation, as suggested by the significant prevalence of denture use in this studied edentulous population.

The increased levels of the inflammatory markers IL-6 and CRP that parallel the severity of periodontal disease in association with higher incidence of COPD-related events support a potential systemic origin of the inflammation and, additionally, also strongly suggest that edentulism is a relevant factor in the morbidity of inflamed COPD patients.

Our study also indicated that while severe periodontal disease was not significantly associated with COPD-related events among study participants classified as GOLD stage III/IV, it was significantly associated among those study participants with GOLD stage I or II disease, even when adjusting for confounders (Table 5). However, when either serum IL-6 or CRP was added to the model, severe periodontal disease was no longer significantly associated (Table 7).

One reason this may have occurred is that the majority of tooth loss can be attributed to two conditions; dental caries and periodontal disease, with periodontal disease tending to increase with age and being responsible for the majority of tooth loss in a sub-group of the population. Periodontal disease has been associated with a variety of systemic, inflammatory conditions and there is evidence to indicate that components associated with host immune and inflammatory responses as well as systemic exposure to oral pathogens are involved [22], [23]. Other studies have shown that treating periodontal disease results in reduction of serum inflammatory biomarkers, indicating that periodontal disease may enhance systemic inflammation [24], [25]. Thus, if periodontal disease and these serum biomarkers share the same causal pathway, it was not surprising that severe periodontal disease was no longer significant in individuals in the highest two quartiles of serum IL-6 when being edentulous remained significant.

Our study design may be another potential reason that severe periodontal disease did not remain significant in the model. The oral examination was conducted only at the baseline of this study and periodontal status may have changed. For example, study participants may have sought treatment as a result of the examination and their periodontal status improved; or their periodontal status may have deteriorated and they lost all their teeth and became edentulous; or participants who were healthy at baseline may have acquired periodontal disease. Any of these changes in periodontal status would have resulted in misclassification of the exposure leading to bias towards the null. Conversely, edentulous status would not have changed and would have resulted in minimal, if any, misclassification of that potential exposure.

An additional important contribution of this study is that after adjusting for confounders including BMI, smoking status, race, gender, hypertension and diabetes, IL-6 and CRP, which are two key markers of chronic inflammation, showed association with oral health conditions.

It also should be noted that this study sample is predominately a middle age to older group [mean age 63.9 years old (SD = 5.69)]. Thus the incident rates of COPD-related events are higher than would be found in the general US population. For example, the incidence of hospitalization due to COPD during 2005 was estimated to be 23.6 per 10,000 [26], while Table 1 reports the incidence of COPD-related events to be 24.4 per hundred individuals in this study. In addition to this being an older group, everyone in this study had at least GOLD stage I disease. Thus, the associations shown in this study may not be representative of the entire US population.

In summary, the results of this population based study showed that edentulism, which reflects a complex pattern of environmental, pathogenic and socioeconomic conditions, is predictive of COPD-related events and thus provides further evidence supporting the notion that poor oral health is an important public health issue.

## References

[1] Corbridge S, Wilken L, Kapella MC, Gronkiewicz C (2012) An evidence-based approach to COPD: part 1. Am J Nurs 112: 46–57; quiz 59,58. 22333971 10.1097/01.NAJ.0000412639.08764.21

[2] Gan WQ, Man SF, Senthilselvan A, Sin DD (2004) Association between chronic obstructive pulmonary disease and systemic inflammation: a systematic review and a meta-analysis. Thorax 59: 574–580. 15223864 10.1136/thx.2003.019588 PMC1747070

[3] Buist AS, McBurnie MA, Vollmer WM, Gillespie S, Burney P, et al (2007) International variation in the prevalence of COPD (the BOLD Study): a population-based prevalence study. Lancet 370: 741–750. 17765523 10.1016/S0140-6736(07)61377-4

[4] Rabe KF, Hurd S, Anzueto A, Barnes PJ, Buist SA, et al (2007) Global strategy for the diagnosis, management, and prevention of chronic obstructive pulmonary disease: GOLD executive summary. Am J Respir Crit Care Med 176: 532–555. 17507545 10.1164/rccm.200703-456SO

[5] Azarpazhooh A, Leake JL (2006) Systematic review of the association between respiratory diseases and oral health. J Periodontol 77: 1465–1482. 16945022 10.1902/jop.2006.060010

[6] Scannapieco FA, Bush RB, Paju S (2003) Associations between periodontal disease and risk for nosocomial bacterial pneumonia and chronic obstructive pulmonary disease. A systematic review. Ann Periodontol 8: 54–69. 14971248 10.1902/annals.2003.8.1.54

[7] Preshaw PM, Walls AW, Jakubovics NS, Moynihan PJ, Jepson NJ, et al (2011) Association of removable partial denture use with oral and systemic health. J Dent 39: 711–719. 21924317 10.1016/j.jdent.2011.08.018

[8] Barros SP, Al-Tarawneh SK, Bencharit S, Loewy Z, Gendreau L, et al (2012) Salivary Cytokines and C. albicans levels in Denture Stomatitis: an Exploratory Case-Control study. OJST

[9] Glass RT, Conrad RS, Bullard JW, Goodson LB, Mehta N, et al (2010) Evaluation of microbial flora found in previously worn prostheses from the Northeast and Southwest regions of the United States. J Prosthet Dent 103: 384–389. 20493328 10.1016/S0022-3913(10)60083-2

[10] Karadag F, Kirdar S, Karul AB, Ceylan E (2008) The value of C-reactive protein as a marker of systemic inflammation in stable chronic obstructive pulmonary disease. Eur J Intern Med 19: 104–108. 18249305 10.1016/j.ejim.2007.04.026

[11] Sin DD, Man SF (2008) Interleukin-6: a red herring or a real catch in COPD? Chest 133: 4–6. 18187736 10.1378/chest.07-2085

[12] Zandvoort A, van der Geld YM, Jonker MR, Noordhoek JA, Vos JT, et al (2006) High ICAM-1 gene expression in pulmonary fibroblasts of COPD patients: a reflection of an enhanced immunological function. Eur Respir J 28: 113–122. 16611655 10.1183/09031936.06.00116205

[13] Mannino DM, Doherty DE, Sonia Buist A (2006) Global Initiative on Obstructive Lung Disease (GOLD) classification of lung disease and mortality: findings from the Atherosclerosis Risk in Communities (ARIC) study. Respir Med 100: 115–122. 15893923 10.1016/j.rmed.2005.03.035

[14] Agusti AG (2005) COPD, a multicomponent disease: implications for management. Respir Med 99: 670–682. 15878483 10.1016/j.rmed.2004.11.006

[15] Campos MS, Marchini L, Bernardes LA, Paulino LC, Nobrega FG (2008) Biofilm microbial communities of denture stomatitis. Oral Microbiol Immunol 23: 419–424. 18793366 10.1111/j.1399-302X.2008.00445.x

[16] Budtz-Jorgensen E, Stenderup A, Grabowski M (1975) An epidemiologic study of yeasts in elderly denture wearers. Community Dent Oral Epidemiol 3: 115–119. 1056815 10.1111/j.1600-0528.1975.tb00291.x

[17] Arendorf TM, Walker DM (1987) Denture stomatitis: a review. J Oral Rehabil 14: 217–227. 3298586 10.1111/j.1365-2842.1987.tb00713.x

[18] Figueiral MH, Azul A, Pinto E, Fonseca PA, Branco FM, et al (2007) Denture-related stomatitis: identification of aetiological and predisposing factors - a large cohort. J Oral Rehabil 34: 448–455. 17518980 10.1111/j.1365-2842.2007.01709.x

[19] Shulman JD, Beach MM, Rivera-Hidalgo F (2004) The prevalence of oral mucosal lesions in U.S. adults: data from the Third National Health and Nutrition Examination Survey, 1988–1994. J Am Dent Assoc 135: 1279–1286. 15493392 10.14219/jada.archive.2004.0403

[20] Vanden Abbeele A, de Meel H, Ahariz M, Perraudin JP, Beyer I, et al (2008) Denture contamination by yeasts in the elderly. Gerodontology 25: 222–228. 18665849 10.1111/j.1741-2358.2007.00247.x

[21] Quirynen M, Van Assche N (2011) Microbial changes after full-mouth tooth extraction, followed by 2-stage implant placement. J Clin Periodontol 38: 581–589. 21488934 10.1111/j.1600-051X.2011.01728.x

[22] Andriankaja OM, Barros SP, Moss K, Panagakos FS, DeVizio W, et al (2009) Levels of serum interleukin (IL)-6 and gingival crevicular fluid of IL-1beta and prostaglandin E(2) among non-smoking subjects with gingivitis and type 2 diabetes. J Periodontol 80: 307–316. 19186972 10.1902/jop.2009.080385

[23] Offenbacher S, Beck JD, Moss K, Mendoza L, Paquette DW, et al (2009) Results from the Periodontitis and Vascular Events (PAVE) Study: a pilot multicentered, randomized, controlled trial to study effects of periodontal therapy in a secondary prevention model of cardiovascular disease. J Periodontol 80: 190–201. 19186958 10.1902/jop.2009.080007 PMC2778200

[24] Koromantzos PA, Makrilakis K, Dereka X, Offenbacher S, Katsilambros N, et al (2012) Effect of non-surgical periodontal therapy on C-reactive protein, oxidative stress, and matrix metalloproteinase (MMP)-9 and MMP-2 levels in patients with type 2 diabetes: a randomized controlled study. J Periodontol 83: 3–10. 21627458 10.1902/jop.2011.110148

[25] Elter JR, Hinderliter AL, Offenbacher S, Beck JD, Caughey M, et al (2006) The effects of periodontal therapy on vascular endothelial function: a pilot trial. Am Heart J 151: 47. 16368290 10.1016/j.ahj.2005.10.002

[26] Brown DW, Croft JB, Greenlund KJ, Giles WH (2010) Trends in hospitalization with chronic obstructive pulmonary disease-United States, 1990–2005. COPD 7: 59–62. 20214464 10.3109/15412550903499548

