# Correction: A Cohort Study of the Impact of Tooth Loss and Periodontal Disease on Respiratory Events among COPD Subjects: Modulatory Role of Systemic Biomarkers of Inflammation

**DOI:** 10.1371/annotation/066cd415-327b-41cb-adce-3db7e5cb527d

**Published:** 2013-08-26

**Authors:** Silvana P. Barros, Robert Suruki, Zvi G. Loewy, James D. Beck, Steven Offenbacher

The third author, Zvi G. Loewy, was given the incorrect affiliation. The correct affiliations for this author are:

Department of Pharmaceutical and Biomedical Sciences, Touro College of Pharmacy, New York, New York, United States of America

and

Department of Biochemistry and Molecular Biology, New York Medical College, Valhalla, New York, United States of America 

